# Redescription of an endemic mahseer, *Tor mahanadicus* (David, 1953) from Mahanadi River basin, India based on integrated morphological and molecular techniques

**DOI:** 10.1371/journal.pone.0291436

**Published:** 2023-09-12

**Authors:** Jeyaraj Antony Johnson, Prasanta Nanda, Bhawana Pant, Sneha Shivaji Mane, Vishnupriya Kolipakam

**Affiliations:** 1 Wildlife Institute of India, Dehradun, Uttarakhand, India; 2 Department of Zoology, Dera Natung Government College, Itanagar, Arunachal Pradesh, India; Sher-e-Kashmir University of Agricultural Sciences and Technology of Kashmir, INDIA

## Abstract

*Tor mahanadicus* was originally described as *Tor mosal mahanadicus* with inadequate information on its morphological traits and no designated type specimen. Currently, *T*. *mahanadicus* is synonymized with *Tor putitora*, solely based on partial molecular data despite significant morphological differences. In this study, we performed an integrated morphological and molecular analysis to redescribe *T*. *mahanadicus* from the Mahanadi River. *Tor mahanadicus* is distinguished from all known Indian *Tor* species by the presence of 2 complete rows of scales between pelvic fin origin and lateral line, small eye (15.3–16.9% in head length), and a wide mouth gap (21.7–23.8% in head length). Moreover, it undoubtedly distinguished from the closely related species *Tor putitora* by having a wider body depth (26.8–28.2% in standard length vs. 14.5–19.9%), short snout length (23.3–26.4% in head length vs. 28.0–29.3%) and wide inter orbit space (30.5–37.3% in head length vs. 27.6–28.5%). Additionally, the molecular phylogenetic tree generated from a combination of three genes demonstrates a monophyletic clade separate from the *Tor putitora* clade. Based on the distinct morphological traits and mitochondrial gene sequences, we established *Tor mahanadicus* as separate species under the genus *Tor*.

## 1 Introduction

Mahseers are large-sized cyprinids (Cypriniformes: Cyprinidae: Torinae) belonging to the genus *Tor* and *Neolissochilus*, which thrive in the fast-flowing rivers of India and the Indian subcontinent. The genus *Tor* comprises a diverse group of mahseer species, with eight valid species recognized in Indian waters [1]. However, taxonomic ambiguity remains in the *Tor* genus due to poor diagnostic characters and vague original descriptions of many mahseer species [2]. One such species is *Tor mahanadicus*, found only in the Mahanadi River in Odisha. *Tor mahanadicus* was originally described by David in 1953 [3] from the Hirakud stretch of the Mahanadi River as *Tor mosal mahanadicus*. According to David [3], it differs from *Tor mosal* by narrow body depth (body depth is shorter than the head length vs. equal head length and body depth ratio), small eye (eye diameter more than 4.5 times in head length vs. less than 4.5 times) and body with a light grey band running from opercle to the caudal fin base vs. no such lateral band on the body [3]. Other morphometric characters, such as lateral transverse scale rows, preanal scales, circumferential scales, etc., were not described in the original description.

Further, no type specimen was designated for this species [3]. Despite distinct morphological features, validity of this species was debated due to incomplete information in the original description. Menon in 1992 [4] synonymized this species with *Tor tor* based on the close similarity in head length and body depth in standard length. In this context, the first molecular study was carried out to discriminate five mahseers, including *Tor mahanadicus*, by Mohindra et al. [5] and concluded that *Tor mahanadicus* and *Tor putitora* were closely related based on the RAPD profile of 80 loci genes. Later, Khare et al. [6] used a partial sequence of d-loop and COI genes to demonstrate that *Tor mahanadicus* is synonymous with *Tor putitora* due to their interpretation of the lack of genetic differences between *Tor mahanadicus* and *Tor putitora*. However, these studies did not consider other morphometric characters or additional genes, and the species status of *Tor mahanadicus* has been compromised with the Himalayan mahseer *Tor putitora* based solely on the similarity of partial mitochondrial sequence [6]. So far, the natural distribution of *Tor putitora* is known from the Himalayan river systems, whereas *Tor mahanadicus* is known only from the Mahanadi River basin in the Deccan plateau [3, 4, 7].

Similarly, there are many notable morphological differences occur between *Tor putitora* and *Tor mahanadicus*, including head size, body depth, transverse scale counts, and body coloration. As a result of this synonymy of *T*. *putitora* and *T*. *mahanadicus*, the geographical distribution range of *T*. *putitora* has been extended to the Central and Eastern Ghats region. Thus, the conservation status of *Tor putitora* has been compromised due to the wide geographical distribution of species. Both the molecular studies on Mahseer [5, 6] have mainly relied on partial gene sequences without examining the morphological traits of *Tor putitora* and *Tor mahanadicus*. It is, therefore, prudent to reevaluate the status of *Tor mahanadicus* by integrating morphology and molecular techniques. In this context, the present investigation is aimed to redescribe and to clarify taxonomic status of *Tor mahanadicus* based on a detailed analysis of morphological characters coupled with more robust mitochondrial sequence data.

## 2 Methods

Fish samples were obtained from the local fishermen, who have been fishing for their livelihood at different sectors of the Mahanadi River, Odisha. Fishes were directly purchased from the fishermen at the local landing area in Koligoghar (KG–about 80 km upstream of Hirakud, Lat. 21°48’19”N and Long. 83°40’06”E), Huma (HD–about 40 km below the Hirakud, where the *Tor mahanadicus* was originally described, Lat. 21°16’49”N and Long. 83°54’40”E), Binka (BN–about 80 km below the Hirakud, Lat. 21°00’29”N and Long. 83°49’08”E) and Boudh (about 120 km from Hirakud, Lat. 21°49’39”N and Long. 84°21’16”E). Subsequently, we obtained *Tor putitora* from fisherman, who were fishing in Kosi and Kollu rivers of the Ramaganga River basin, Uttarakhand, and *Tor tor* specimens from the Chambal River at Kota, Rajasthan. After obtaining fish catch from the fisherman, the specimens were photographed, and a portion of one side of the pelvic fin was clipped from each specimen and preserved in 95% high gradient ethanol. The specimens were then preserved in 10% formalin, and some quantity of formalin was injected inside the abdomen, operculum, and body muscle for proper preservation. Since, the study did not involve animal sacrifice, the ethical approval is not applicable. Fish morphometric and meristic measurements were taken [8, 9]. Dial calipers with an accuracy of 0.01 mm were used to make morphometric measurements, while magnifying head lenses and a dissection microscope were employed to count meristic characters. Morphometric characters associated with body were converted into percentage in standard length (SL) and characters associated with head were converted into percentage in head length (HL).

### 2.1 Molecular characterization

Genomic DNA was extracted using DNeasy Blood and Tissue Kit (Qiagen, Hilden, Germany) to target the COI (700 bp), complete *cytb* (1140 bp), and control region gene (940 bp) for PCR amplification. The primer pairs L14724/H15915 [10], DL1/DH2 [11], and fish-specific primers Fish F1/R1 [12] were used to amplify the *cytb*, control region, and COI, respectively. The PCR reaction mixture contained 5 μL of PCR Mastermix (Qiagen), 2 pmol of each forward and reverse primer, 1.2 μl of BSA (2μM), and 1μl of template DNA in a total volume of 15 μl. The PCR conditions involved an initial denaturation at 95°C for 15 minutes, followed by 35 cycles of denaturation at 95°C for 45 seconds, annealing at 55°C for 45 seconds, extension at 72°C for 1 minute, and final extension at 72°C for 10 minutes. Positive and negative controls were also included. PCR products were visualized using SYBr Safe staining on a 2% agarose gel under UV light. PCR products were treated with Exonuclease I (EXO-I) and shrimp alkaline phosphatase (SAP) for 15 minutes each at 37°C and 80°C to remove residual primers, followed by sequencing of both directions using the BigDye® Terminator Kit (v3.1) and an ABI 3500XL Applied Biosystems Genetic Analyzer.

### 2.2 Sequence analysis

The DNA sequences were edited using Chromas and aligned with ClustalW from MEGA X [13]. The generated sequences were deposited in Genbank (Accession numbers OQ674797—OQ674804 & OQ658608—OQ658613). CR and *cytb* sequences previously reported in the public NCBI database were retrieved for the final phylogenetic analysis. *Puntius titteya*, and *Systomus sarana* were used as outgroup species. The genetic distance between species groups was estimated in MEGA X [13] using p-distance, with a bootstrap of 1000 iterations. Maximum Likelihood (ML) analysis was performed for cytochrome b nad controle region im MEGA X [13]. The optimal partition scheme and evolutionary model were determined for each locus using IQ-tree 2.0.4 [14] and the Bayesian inference (BI) criterion. The genetic distance index, termed as p-distance, using the combined gene sequences between species groups was measured using MEGA X [13].

The results suggested that the best-fit model for the dataset is the general time reversible substitution model with a gamma distribution of rates (GTR+G). We used BEAUTi and Bayesian Evolutionary Analysis by Sampling Trees (BEAST2) software package v.2.4.4 to construct a coalescent phylogeny [15]. Based on the strict molecular clock, the Bayesian phylogenetic inference was implemented through Markov Chain Monte Carlo (MCMC) simulations [16]. The input data for BEAST2 was a concatenated alignment of 1928 bp in FASTA format. We performed 15 independent chains for 100 million generations, with a random starting tree, and sampled every 1,000 generations. The initial 25% of generations were discarded as burn-in. The convergence of MCMC chains was visually checked, and the effective sample size (ESS) was found to be above 200 by exploring the likelihood plots in TRACER v1.7 [17]. TREEANNOTATOR was used to summarize posterior parameters from tree samples and to generate a maximum clade credibility tree, visualized with FigTree v.1.4.3 [18].

## 3 Results

### 3.1 Diagnosis

*Tor mahanadicus* can be distinguished from all known Indian *Tor* species based on the presence of the following characteristics: 2 rows of scale between pelvic-fin origin and lateral line; mouth gap extended below the anterior margin of eye orbit. Furthermore, it also distinguished from congeneric species by the following combination of characters: presence of small eye (eye diameter 15.3–16.9% in HL), short snout length (23.3–26.4% in HL), and wide inter orbit distance (30.5–37.3% in HL).

### 3.2 Description

**Fig 1:**
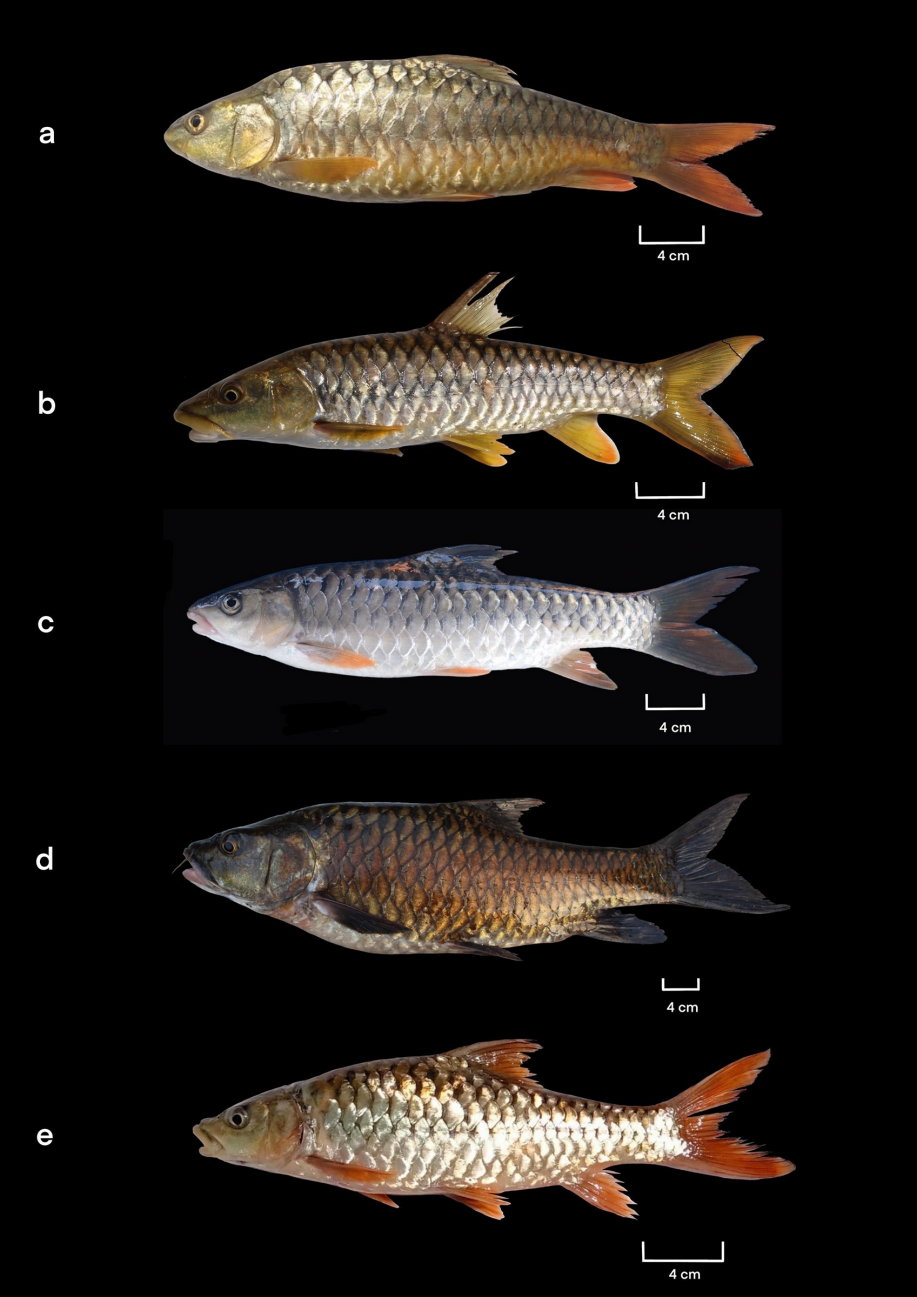
(a). Lateral view of *Tor mahanadicus*, 31.7 cm SL from Type locality, Sampalpur, River Mahanadi, Odisha, Photo credit: Prasanta Nanda; (b). *Tor putitora*, 29.5 cm SL from Kollu river, Ramaganga River basin, Uttarakhand, Photo credit: J.A. Johnson; (c). *Tor mosal*, 31 cm SL from Jia Bhorali, Assam, Photo credit: Boni Amin Laskar; (d). *Tor khudree*, 50 cm SL from Koyna Reservoir, Krishna River basin, Maharastra, Photo credit: J.A. Johnson; (e), *Tor tor*, 24.5 cm SL from River Chambal, Rajasthan, Photo credit: J.A. Johnson.

The general body shape of *Tor mahanadicus* is depicted in Fig 1A, and morphometric data of voucher specimens is summarized in Table 1. Body short and laterally compressed, its depth 26.8–28.2% in SL and width 25.1–27.1% in SL. Dorsal and ventral profile of anterior body equally arched and profile shape sharply drop at posterior portion from the dorsal-fin origin to anal-fin origin. Head small, its length 23.7–26.9% in SL. Dorsal profile of head sharply declines at snout. Eyes small, dorso-laterally placed and visible from ventral side, its diameter 15.3–16.9% in HL and inter-orbital distance 30.5–37.3% in HL. Snout short, its length 23.3–26.4% in HL. No tubercles on snout and chin. Mouth subterminal, wide, opening extends below the front portion of the eyes and mouth gap 21.7–23.8% in HL. Upper jaw with a deep free space in between, its length 26.6–28.9% in HL and lower jaw short, its length 17.0–18.2% in HL. Barbels two pairs; rostral barbel located antero-lateral position, size equal to eye diameter, its length 15.3–17.6% in HL and maxillary barbel located at the corner of the mouth, originating deep behind the rostral cap. Maxillary barbels longer than rostral barbel, its length 17.7–25.4% in HL. Nostril located closer to the anterior margin of the eye than to the tip of the snout, and inter-narial distance 18.8–23.5% in HL. Nares are separated by a small rounded membranous flap, which divides the nostrils into anterior and posterior nare with equal size.

**Table 1:** Morphometric measurements of *Tor mahanadicus*.

Characters	*Tor mahanadicus* (n = 5)	
	Range	Mean ± SE
Standard length (mm)	230–317	**-**
**% in Standard length**		
Head length	23.7–26.9	25.8 ± 0.74
Head width	13.9–15.4	14.6 ± 0.31
Body depth	26.8–28.2	27.8 ± 0.34
Body width	25.1–27.1	26.5 ± 0.46
Pre dorsal	45.9–52.2	49.5 ± 1.41
Pre-pectoral	22.3–28.7	24.6 ± 1.41
Pre pelvic	46.7–52.4	49.3 ± 1.45
Pre anal	69.3–82.9	76.3 ± 3.58
Length of dorsal fin	19.5–22.3	20.4 ± 0.66
Length of pectoral fin	17.6–20.7	18.6 ± 0.71
Length of pelvic fin	15.3–17.4	16.2 ± 0.45
Length of anal fin	16.8–20.3	18.4 ± 0.72
Length of caudal fin	23.5–27.5	24.7 ± 0.94
Length of caudal peduncle	15.3–17.4	16.3 ± 0.46
Depth of caudal peduncle	10.8–12.5	11.6 ± 0.35
Distance pectoral and anal fin	18.9–26.1	22.8 ± 1.49
Distance pelvic and anal fin	24.6–28.7	26.4 ± 0.85
Height of CP/ Length of CP	67.4–70.2	69.2 ± 0.65
**% in Head length**		
Eye diameter	15.3–16.9	15.9 ± 0.37
Inter orbital distance	30.5–37.3	34.5 ± 1.45
Inter narial distance	18.8–23.5	20.9 ± 1.06
Snout length	23.3–26.4	24.6 ± 0.66
Pre orbital distance	32.4–36.3	34.3 ± 0.85
Pre nasal distance	24.6–26.4	25.7 ± 0.41
Mouth gap	21.7–23.8	22.6 ± 0.46
Length of rostral barbel	15.3–17.6	16.8 ± 0.50
Length of Maxillary barbel	17.7–25.5	21.5 ± 1.60
Upper jaw length	26.6–28.9	28.1 ± 0.51
Lower jaw length	17.0–18.2	17.8 ± 0.27

Dorsal fin with 4 simple and 9 branched rays; last unbranched ray strong and osseous, and its length 19.5–22.3% in SL. Dorsal fin inserted at middle of the body, vertically just opposite to pelvic-fin origin with a predorsal distance of 45.9–52.2% in SL. The anterior margin of dorsal fin slightly convex towards the tip, the posterior one straight, distal slightly concave up to the fourth branched rays, and then obliquely straight. Dorsal fin length slightly longer than pectoral-fin length, and its length 19.5–22.3% in SL. Pectoral fin short, reaches up to 5th lateral line scale, its length 17.6–20.7% in SL and it contains 1 simple and 12 to 13 branched rays and placed laterally with pre-pectoral length of 22.3–28.7% SL. Pelvic fin small, originated at vertically just opposite to origin of dorsal fin with pre-pelvic distance of 46.7–52.4% in SL and its length 15.3–17.4% in SL. Pelvic fin contains 1 unbranched and 8 branched rays and the tip of pelvic fin not reaching anus. The anal fin composed of 1 simple and 6 branched rays; last branched ray well-branched; the anterior fin margin deeply convex towards the tip and outer margin of the fin straight. Anal fin inserted with preanal distance of 69.3–82.9% in SL. Lateral line complete, straight medially, with slight concavity at the anterior end, with 26 scales, scales perforated and large. Transverse scale rows between the lateral line and dorsal-fin origin 3.5 and 2 scale rows present between the lateral line and pelvic-fin origin. The circumferential region contains 18 scales. Caudal peduncle short, and its height 67.4–70.2% in caudal peduncle length, and the curcumpeduncular region contains 12 scale rows. Caudal fin deeply forked with the first 1 simple ray, 8+9 branched rays, lobes equal in size with pointed tip.

#### Colour

In fresh specimen, dorsal and lateral side of the body up to lateral line grey colour with golden shade on scales. Lateral side below lateral line, and ventral surface golden yellow. A dark brown indistinct brown band runs from opercula to caudal fin base (Fig 1A). Head and chin golden yellow. Dorsal greenish yellow, rest all fins (pectoral, pelvic, anal, and caudal fins) deep orange colour. In formalin preservation, dorsal side of head and body dark grey, and grey extends up to lateral line. Below, lateral line and ventral region white. The dorsal fin dark grey, and caudal fin light grey colour. Rest of the fins (pectoral, pelvic, and anal fins) white.

### 3.3 Genetic distance and molecular phylogeny

The p-distance values obtained for *Tor mahanadicus* and other closely related species are given in Table 2. The analysis was run separately for control region, *cytb*, and COI, as well as combined analysis was run with different patterns to obtain a combined species tree. Since the COI region did not have many polymorphic sites, and comparable data between the present study and Khare et al. [6] or other sequences were minimal, this further reduced the robustness of inference. Therefore, the remaining part of the analysis was with the control region and *cytb*. The control region sequence is specifically known to help delineate species in Cyprinidae and therefore presents a more relevant result than other genes [11, 19]. The p-distance calculated between species for *Tor tor* and *Tor mahanadicus* is 0.031, and between *Tor putitora* and *Tor mahanadicus* is 0.006, further *Tor mahanadicus* clearly separated from *Tor mosal* with genetic distance of 0.06. In the ML and Bayesian phylogenetic analysis of combined genes of the control region and *cytb*, the samples collected from the Mahanadi River (BN—Binika, HD—Huma, and KG—Koligoghar) formed a monophyletic and highly supported clade (PP ~1), showing that *Tor mahanadicus* is clearly distinct. The four samples of *Tor putitora* collected from Kosi and Kollu rivers, Uttarakhand, clustered into a clade with (PP~0.99).

**Table 2:** Genetic distance between closely related *Tor* species (values generated based on the combined sequence of cytb and control region).

	*T*. *mahanadicus*	*T*. *putitora*	*T*. *mosal*	*T*. *tor*	*T*. *khudree*
*T*. *putitora*	0.006				
*T*. mosal*	0.060	0.058			
*T*. *tor*	0.031	0.031	0.052		
*T*. *khudree*	0.072	0.073	0.036	0.066	
*T*. *barakae*	0.072	0.069	0.005	0.063	0.051

*only *cytb* gene is used for generating genetic distance

**Fig 2:**
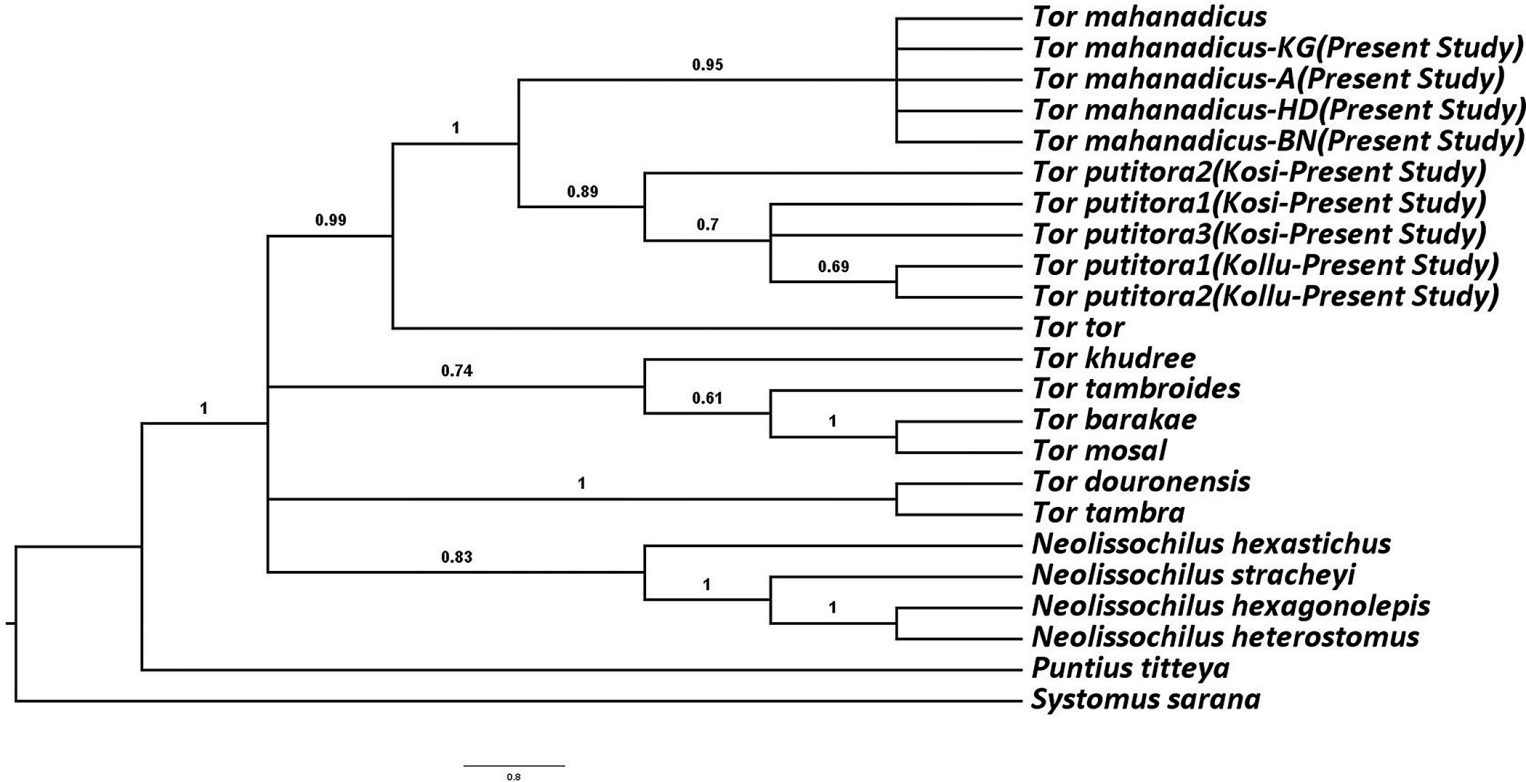
ML tree of *Tor* species based on mtDNA cytb with bootstrap support values.

**Fig 3:**
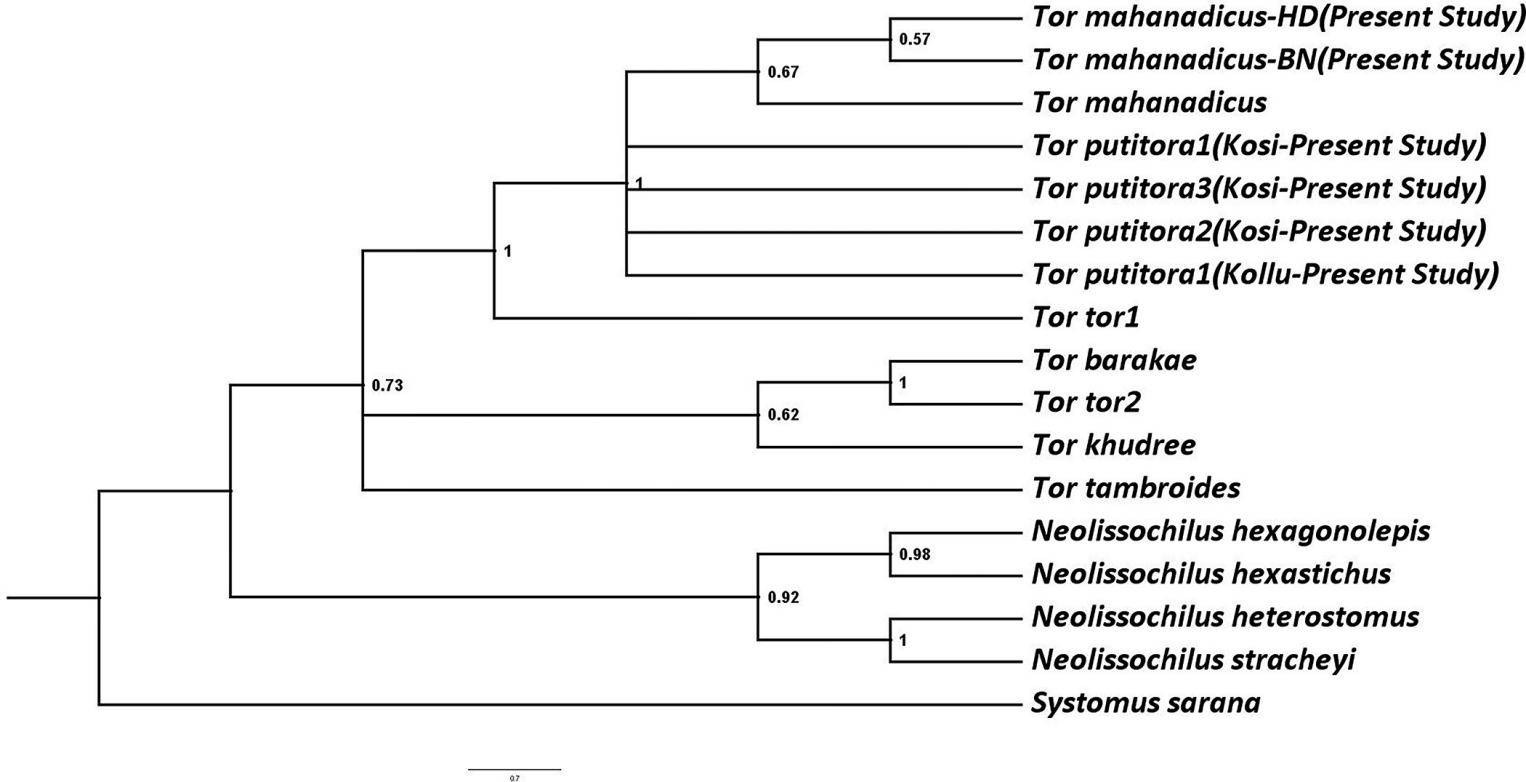
ML tree of *Tor* species based on control region with bootstrap support values.

**Fig 4:**
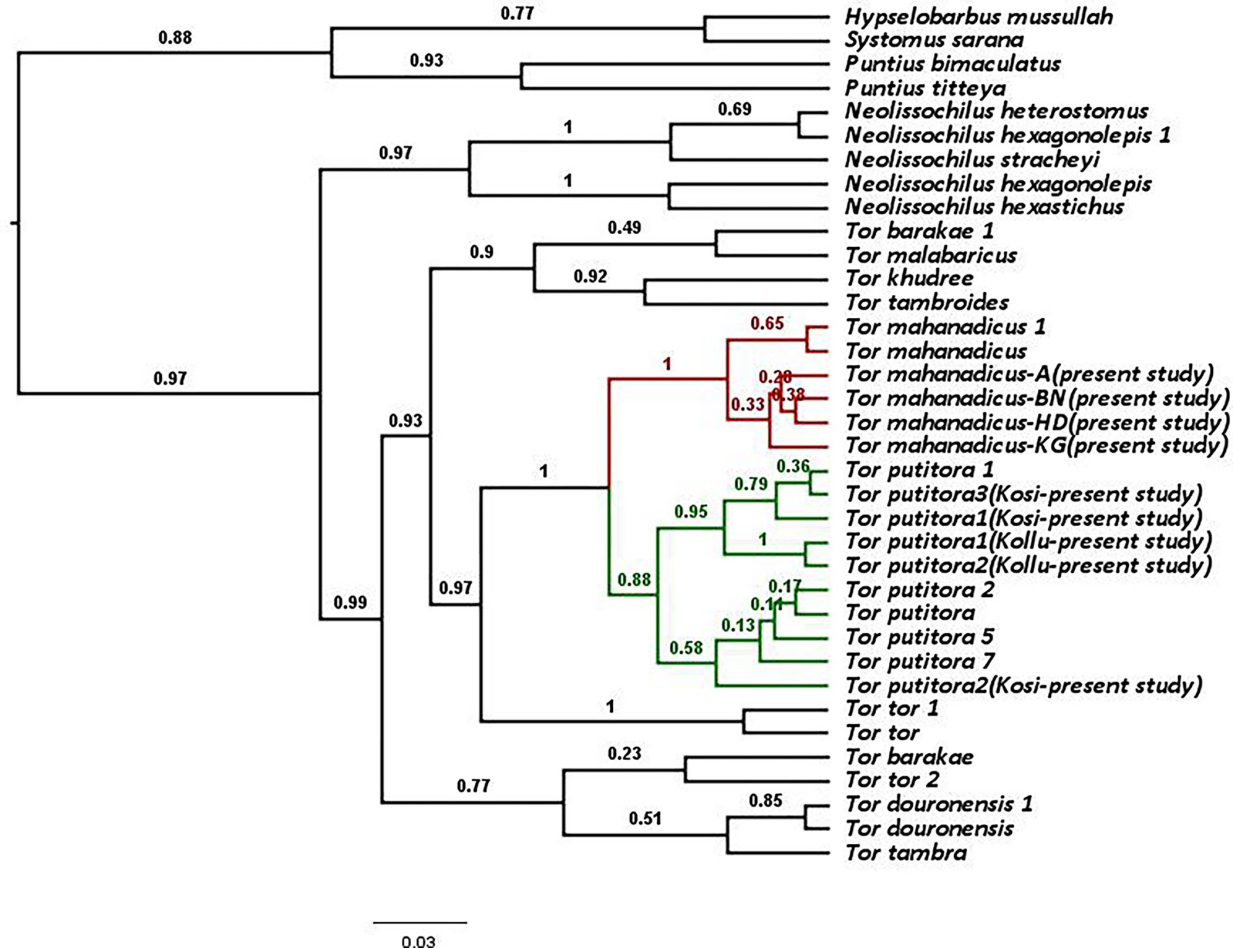
Bayesian tree of *Tor* spp. based on the mtDNA cytb and control region. Posterior probability values are provided at their respective nodes. The clade of *T*. *putitora* is marked in green, while *T*. *mahanadicus* is marked in red.

The ML tree based on *cytb* and control region (Figs 2 and 3) revealed that *Tor mahanadicus* diverged very well from the *Tor tor* clade. Both ML and Bayesian Analysis yielded the same grouping of monophyly for *Tor mahanadicus* and all trees contained the same major clades, but the branching order was different (Figs 2–4).

### 3.4 Material examined

*Tor putitora*, WII/F-301, 2ex., 29.5 and 16.4 cm SL, Kollu River, Ramagang River basin, Uttarakhand, India. *Tor putitora*, WII/F-302, 1 ex., 69 cm SL, Yamuna River, Dakpakta Barrage, Uttarkhand. *Tor tor*, WII/F-303-305, 3 ex., 23.5, 24, 24.5 cm SL, Chambal River, Rajasthan. *Tor khudree*, WII/F-306-309, 4 ex, 18.5, 17, 16.8, 13.2 cm SL, Koyna Reservoir, Krishna River basin, Maharastra. *Tor mosal*, BMF-4431 cm SL, Jia Bhorali, Assam. *Tor moasl*, ZSI/FF-3347, 28 cm SL, Tuirial River, Mizoram (ZSI digital collection).

## 4 Discussion

The identity of Mahanadi mahseer, *Tor mahandicus* has been compromised with other mahseer by different authors based on one or two identical morphological traits. Menon [4] considered *Tor mahanadicus* as synonym of *Tor tor* and *Tor mosal* a junior synonym of *Tor tor* based on the similarity in head length and body ratio. Recent studies by Mohindra et al. [5] and Khare et al. [6] considered *Tor mahanadicus* as *Tor putitora* based on close similarity in genetic structure. However, the present investigation of detailed morphological traits revealed that *Tor mahanadicus* showing distinct morphological traits with *Tor mosal*, *Tor tor*, *Tor putitora* and other known deccan mahseer *Tor khudree*. The comparison of conspicuous morphological traits of *Tor mahanadicus* with other *Tor* species is presented in Table 3 and their external morphological appearance is presented in Fig 1A–1E. The original description of morphological traits of *Tor mahanadicus* (= *Tor mosal mahanadicus*) was said to be very closely related to *Tor mosal* [3]. Menon [4] has pointed out that the best method to separate between *Tor* species is based on the head length/ body depth ratio. This was clear evidence in the case of the distinction between *T*. *mahanadicus* and *T*. *mosal*. According to David [3], the main distinction between *T*. *mahanadicus* and *T*. *mosal* is the presence of small head (length of head less than body depth vs. length of head equal to body depth). Further, *T*. *mahanadicus* differs from *T*. *mosal* in other morphological traits: more robust body (body width 25.1–27.1 vs. 11.78% in SL) presence of large eye (eye diameter 15.3–16.9 vs. 11.78% in head length); 2 scales between lateral line and pelvic fin vs. 3 scales). Further, ML tree produced based on the *cytb* (Fig 3) and based on the genetic distance (0.06) between *T*. *mahanadicus* and *T*. *mosal* revealed that *T*. *mahanadicus* is quite district from *T*. *mosal*.

**Table 3:** Comparison of conspicuous morphological traits of *Tor mahanadicus* with other closely related *Tor* species.

Characters	*Tor mahanadicus*	*Tor putitora*	*Tor tor*	*Tor mosal*	*Tor khudree*
Body depth in SL	26.8–28.2	14.5–19.9	24.1–30.3	28.57	22.2–27.3
Body width in SL	25.1–27.1	12.3–14.4	18.0–20.3	11.7	16.2–18.2
Eye diameter in HL	15.3–16.9	18.3–21.6	17.7–19.3	11.7	17.0–21.0
Snout length in HL	23.3–26.4	28.0–29.3	31.4–39.8	29.0	34.0–47.3
Inter orbit width in HL	30.5–37.3	27.6–28.5	34.7–37.8	31.4	29.1–36.8
Transverse scale rows between lateral line and pelvic fin origin	2	2½	2½	3	2½-3
Body profile	Deeply arched dorsal and ventral profile, both equally arched	Body considerably long, almost straight	Dorsal profile deeply arched and ventral profile slightly arched	Dorsal and ventral profile almost straight	Dorsal and ventral profile equally arched
Head length	Less than body depth	Greater than body depth	Less than body depth	Equal to body depth	More or less equal to body depth
Mouth opening	Mouth opening extend below the anterior margin of the eye orbit	Mouth opening reaching below the nares	Mouth opening reaching up to nares	Mouth opening reaching below the nares	Mouth opening reaching Infront of nares
Fin coluor (in live)	Dorsal greenish yellow, all other fins are orange colour	Dorsal greyish, all other fins are golden yellow colour	All fins are bright red colour	Dorsal fin dark grey, Pectoral, Pelvic and Anal fin are red. Caudal fin grey with red shade	All fins are dark blue colour

Meanwhile, the head and body ratio measurement of *T*. *mahanadicus* matches near *T*. *tor* from central Indian rivers, because of that Menon [4] considered *Tor* population form Mahanadi River (*T*. *mahanadicus*) as synonym of *T*. *tor*. However, in the present study, it is observed that *T*. *mahanadicus* differs from *T*. *tor* by many morphometric traits (Table 3): having small eye (15.3–16.9% in HL vs. 17.7–19.3%); short snout length (23.3–26.4% in HL vs. 31.4–39.8%) and presence of 2 scale rows between lateral line and pelvic-fin origin (vs. 2½scales). Further, the phylogenetic trees based on ML and the combined gene Bayesuian tree separates the clades *T*. *mahanadicu*s and *T*. *tor* (Figs 2–4). Therefore, the distinction between *T*. *mahanadicus* and *T*. *tor* is restored. Further, *Tor mahanadicus* differs from other known deccan mahseer *Tor khudree* by robust body (body width 25.1–27.1% in SL vs. 16.2–18.2%); less number of lateral transverse scale (2 vs. 2½-3 scales) and body colour (golden yellow body with red fins vs. dark grey colour with blue fins–Fig 1D). Additionally, the results of genetic analysis revealed that *Tor mahanadicus* is well separated out with genetic distance of 0.072 (Table 2).

Nevertheless, the recent dispute in *Tor* taxonomy in Indian literature is the Khare et al. [6] study, which considered *T*. *mahanadicus* as a synonymy of *Tor putitora*, only based on the partial sequence of COI gene without studying the morphological traits of species. In recent times, there has been a growing trend among researchers to rely solely on molecular markers for determining the species identity without thorough study on the morphometry of voucher specimens [6, 20]. A detailed investigation of morphological traits of *T*. *mahanadicus* and *T*. *putitora* revealed that *T*. *mahanadicus* distinctly differ from the Himalayan mahseer *Tor putitora* (Table 3). *Tor mahanadicus* has been easily distinguished from *T*. *putitora* in many morphometric characters as follows: wider body depth (26.8–28.2% in SL vs. 14.5–19.9%); small eyes (15.3–16.9% in HL vs. 18.3–21.6%); wide inter orbit space (30.5–37.3% in HL vs. 27.6–28.5%); short snout length (23.3–26.4% in HL vs. 28.0–29.3%); deep body (head and body ration 1.73 vs. 0.71) and presence of 2 scale rows between lateral line and pelvic-fin origin (vs. 2½ scales). In addition to the morphological traits, the molecular phylogeny generated from the complete sequence of combined genes of *T*. *mahanadicus* clearly formed a separated cluster out of the *Tor putitora* (Fig 4).

Khare et al. [6] utilized partial sequence of COI and inferred that *Tor mahanadicus* is not distinct from *Tor putitora* because of 0% genetic variation. However, they did not substantiate this conclusion using the complete COI gene along with other markers. Several studies shown that COI as a marker does not perform well for species delimitation for all families of species [19, 21]. Studies have shown that the control region or D-loop marker, employed in the current study, was more informative in resolving the relationships among closely related species within Cyprinidae than other genes [21, 22]. In order to resolve this species validity, in the present study, we have used three genes, control region, *cytb*, and COI genes. The combined results from the *cytb* and control region emphasize that *Tor mahanadicus* monophyly forms a distinct clade with a high posterior probability value (PP~1) (Fig 4) with 0.6% genetic distance. Despite that the average genetic distance between *T*. *mahanadicus* and *T*. *putitora* is low (0.6%), yet *Tor mahanadicus* qualifies as distinct species due to presence of distinct morphotype. Similar low genetic distance (0.8%) was observed between *T*. *mosal* and *T*. *barakae*, in spite of having distinct morphological traits [23]. A study by Yang et al. [24] examined the genetic distances between 35 species in the subfamily Cultrinae, a diverse group of freshwater fish within Cyprinidae and found that the pairwise genetic distance between the species were ranging from less than 1% to 15%. In several species, for example, two species of African cichlid fishes, *Pundamilia pundamilia* and *Pundamilia nyererei*, although these two species have a genetic distance of only 0.4%, they are considered separate species based on their distinct color patterns, breeding behaviours, and ecological niches [25].

Therefore, the present investigation of the integrated and molecular phylogeny of *T*. *mahanadicus* proved that the *T*. *mahanadicus* is a distinct species and completely different from *T*. *putitora* based on distinct morphology and phylogeny. Relying solely on genetic distance [6] to classify species is problematic due to factors such as incomplete lineage sorting, hybridization, and introgression. A more comprehensive approach, including multiple lines of evidence such as morphology, ecology, behavior, and biogeography, is needed to accurately delimit species in fishes and other taxa.

## Supporting information

S1 File (XLSX)
